# Prevalence of Poor Sleep Quality Among Physicians During the COVID-19 Pandemic

**DOI:** 10.7759/cureus.12948

**Published:** 2021-01-27

**Authors:** May M Abdellah, Mohamed F Khalil, Amna Alhafiz

**Affiliations:** 1 Neuropsychiatry Department, Sohag General Hospital, Sohag, EGY; 2 Neurology Department, Saudi German Hospital, Madinah, SAU; 3 Internal Medicine Department, Saudi German Hospital, Madinah, SAU

**Keywords:** sleep, depression, anxiety, physicians, covid-19, pandemic

## Abstract

Background: Sleep quality is influenced adversely or favorably by various intrinsic and extrinsic factors and sleep deprivation is a common problem facing doctors.

Objectives: To assess sleep quality among physicians during coronavirus disease 2019 (COVID-19) pandemic and correlate it with possible predictors.

Methods: This cross-sectional four-months study included a total of 344 physicians from different medical centers in the period between July 2020 and October 2020, during the COVID-19 pandemic. Physicians were aged between 24 and 60 years from different specialties. Data were collected using the Pittsburgh Sleep Quality Index (PSQI) questionnaire and Hospital Anxiety Depression Scale (HADS).

Results: Among our participant physicians there was poor sleep quality in 71.2%, while good sleep quality was present in 28.8%. There were significant correlations between poor sleep quality and the following parameters in the univariate logistic regression analysis: anxiety features (P value <0.001), depressive features (P value <0.001), and past history of COVID-19 (P value 0.003). However, multivariate logistic regression analysis showed that only the presence of anxiety features (P value <0.001) and depressive features (P value <0.001) could be used as significant independent predictor of poor sleep quality among physicians during COVID-19.

Conclusion: Presence of anxiety and or depressive features among physicians are the most significant independent predictors of poor sleep quality during the COVID-19 pandemic.

## Introduction

The World Health Organization declared the global coronavirus disease 2019 (COVID-19) disease outbreak a public health emergency of international concern on January 30, 2020 [1]. Due to the COVID-19 outbreak, measures for social distancing were imposed to control the spread of the pandemic. However, isolation may have negative effect on affect the psychological well-being and sleep quality. Also, changes in family organization and routines of work, plus social isolation, may induce feelings helplessness, insomnia, loneliness, anger, and abandonment [2].

The National Health Commission of China published the National Guideline for Psychological Crisis Intervention for COVID-19, and declared that addressing the mental health repercussions of this epidemic become a nationwide mission that require attention of the whole society. However, no sufficient data were available about the epidemiology and psychological features of communities till now [3].

Fear is an adaptive defense mechanism that is essential for survival and involves several biological processes of preparation for a response to potentially threatening events. However, when it is disproportionate, it can be harmful and may induce several psychiatric disorders. In a pandemic, fear can increase anxiety, stress, and insomnia in healthy individuals and can increase the intensity of symptoms among those with pre-existing psychiatric disorders. In previous epidemic situations, the number of those who have mental health affection tended to be greater than the number of people who were infected by the disease [4].

Healthcare workers are at high risk of developing sleep disorders during an outbreak. Previous studies have addressed the sleep disturbances of healthcare workers during crisis events of public health [5]. Sleep quality has an impact on a physician’s work safety like other people. This is essential for improving the physician health and their daily performance for patient care [6].

Insomnia is a significant public health concern several psychiatric disorders, such as depression and anxiety disorders, which have shown strong relationships with insomnia. However, the clinical impact of the combination of these two conditions on insomnia severity and sleep quality still unclear [7]. Several studies evaluated the health effects of long working hours. Although the results of these studies were inconsistent, many adverse effects on health, such as cardiovascular disease, cognitive impairment and increased incidence of accidents were reported. Insufficient recovery because of sleep deprivation is considered an important component of the pathway leading from long work hours to health problems. Previous studies revealed that working overtime is related to short or disturbed sleep [8]. Literature revealed that developing countries are bearing almost two-thirds of total psychiatric patients in the world, and the situation is expected to worsen [9]. Doctors are at increased risk of carrying mental health challenges which may cause or exacerbate anxiety and depression. Mental health issues of doctors are mostly over-looked not only by public but even by the doctors themselves [10].

This study aimed to assess sleep quality among physicians during the COVID-19 pandemic in some governmental hospitals in Egypt and the Saudi German Hospital-Madinah in the Kingdom of Saudi Arabia, and correlate it with possible predictors to help development of psychological interventions for better quality of life among them during pandemic.

## Materials and methods

This cross-sectional study included 344 physicians from different medical centers in Egypt and the Saudi German Hospital-Madinah, Kingdom of Saudi Arabia in the period between July 2020 and October 2020, during COVID-19 pandemic, with inclusion criteria of physicians aged between 24 and 60 years from different specialties.

Exclusion criteria were: physicians with past history of acute or chronic psychiatric disorders diagnosed by psychiatrist; physicians with past history of acute or chronic medical problems like diabetes mellitus, thyroid disorders, neoplastic diseases, chronic disability, etc.; physicians with current use of medications which may affect psychological state and sleep quality.

All participant physicians were analyzed regarding the followings: age, gender, marital status, past history of COVID-19 infection, and weekly work hours which was categorized into two groups, one group with weekly work hours 36 hours or more and another group with weekly work hours less than 36 hours.

Anxiety and depressive features were assessed among the participant physicians using Hospital Anxiety Depression Scale (HADS), a 14-item self-report measure with seven items forming a depression subscale and another seven constituting an anxiety subscale. Each item is rated on a 4-point scale ranging from 0 to 3, with 3 reflecting the highest distress. The total score for each subscale ranges from 0 to 21 and are categorized as normal (0-7), mild (8-10), moderate (11-14), and severe (15-21) [11]. Sleep quality among the participant physicians was assessed using Pittsburg Sleep Quality Index (PSQI), a self‐report survey evaluating sleep quality and discomfort in the preceding one month. The PSQI consists of a total of 24 questions, 19 of which are based on self‐reports and five of which are evaluated by the individual's spouse or partner. PSQI global scores higher than 5 indicate significantly poor sleep quality [12].

Statistical analysis of the data

Data were fed to the computer and analyzed using Statistical Package for Social Sciences (SPSS) version 20 (IBM Corp., Armonk, NY, USA). The Kolmogorov-Smirnov test was used to verify the normality of distribution of variables; comparisons between groups for categorical variables were assessed using chi-square test (Monte Carlo). Student t-test was used to compare two groups for normally distributed quantitative variables. Significance of the obtained results was judged at the 5% level.

## Results

**Figure 1:**
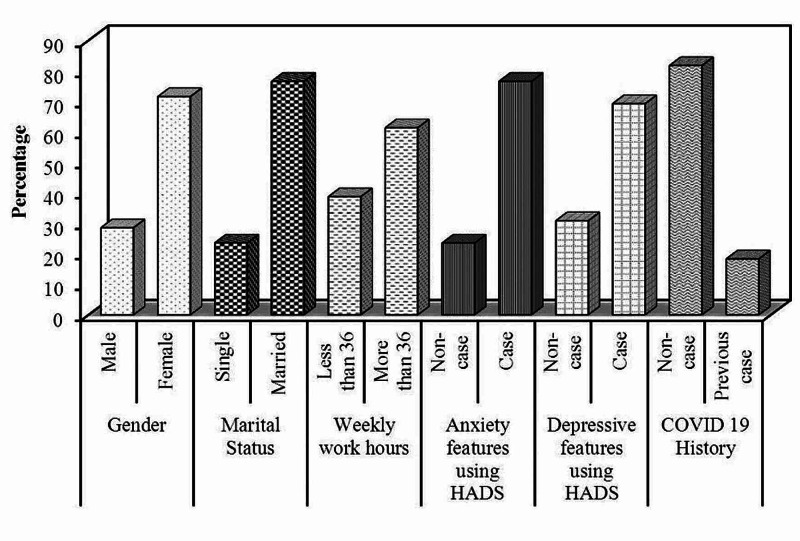
Distribution of demographic data and baseline characteristics among the participant physicians (n = 344) HADS: Hospital Anxiety Depression Scale

**Figure 2:**
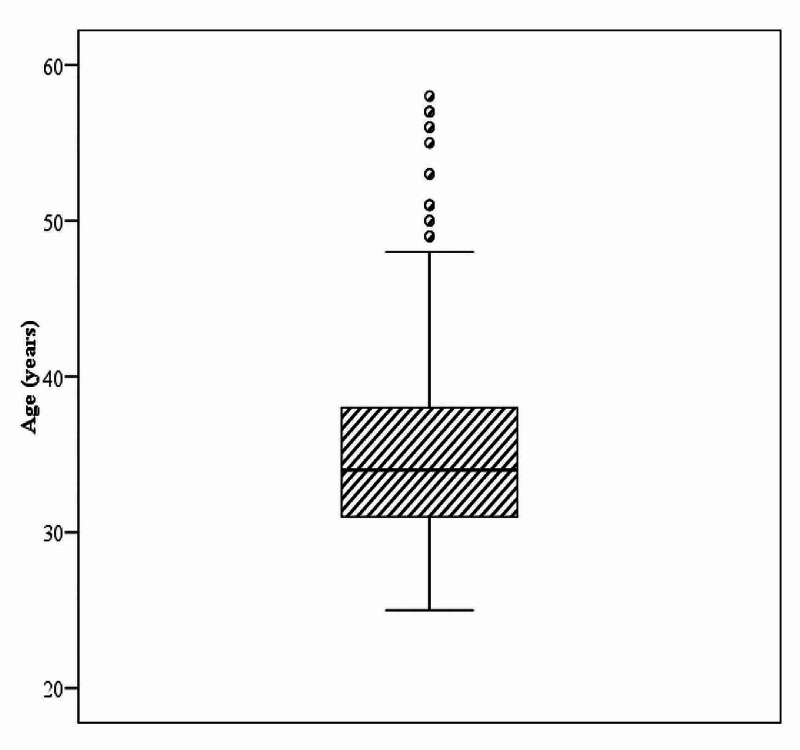
Distribution of age among the participant physicians (n = 344)

Our cross sectional study included 344 physicians. The age of participant physicians ranged between 25 and 60 years with a median age of 34 years; 98 (28.5%) of them were males and 246 (71.5%) of them were females. Also, 81 (23.5%) of our participant physicians were single and 263 (76.5%) of them were married. Regarding the weekly work hours, 133 (38.7%) of them work less than 36 hours per week, while 211 (61.3%) of them work 36 hours or more per week. Among our participant physicians there was previous history of COVID-19 in 63 (18.3%), while 281 (81.7%) had no previous history of COVID-19. Anxiety was present in 263 (76.5%) physicians using HADS, while depression was present in 238 (69.2%) physicians using HADS as shown in Table 1, Figure 1, and Figure 2.

**Table 1:** Distribution of demographic data and baseline characteristics among the participant physicians (n = 344) HADS: Hospital Anxiety Depression Scale; PSQI: Pittsburg Sleep Quality Index

	No. (%)
Age (years)	
Mean ± SD.	35.6 ± 6.7
Median (Min. – Max.)	34 (25 – 60)
Gender	
Male	98 (28.5%)
Female	246 (71.5%)
Marital Status	
Single	81 (23.5%)
Married	263 (76.5%)
Weekly work hours	
Less than 36 hours	133 (38.7%)
36 hours or more	211 (61.3%)
Sleep quality using PSQI	
Poor sleep	245 (71.2%)
Good sleep	99 (28.8%)
Anxiety features using HADS	
Non-case	81 (23.5%)
Case	263 (76.5%)
Depressive features using HADS	
Non-case	106 (30.8%)
Case	238 (69.2%)
COVID 19 History	
Non-case	281 (81.7%)
Previous case	63 (18.3%)

**Figure 3:**
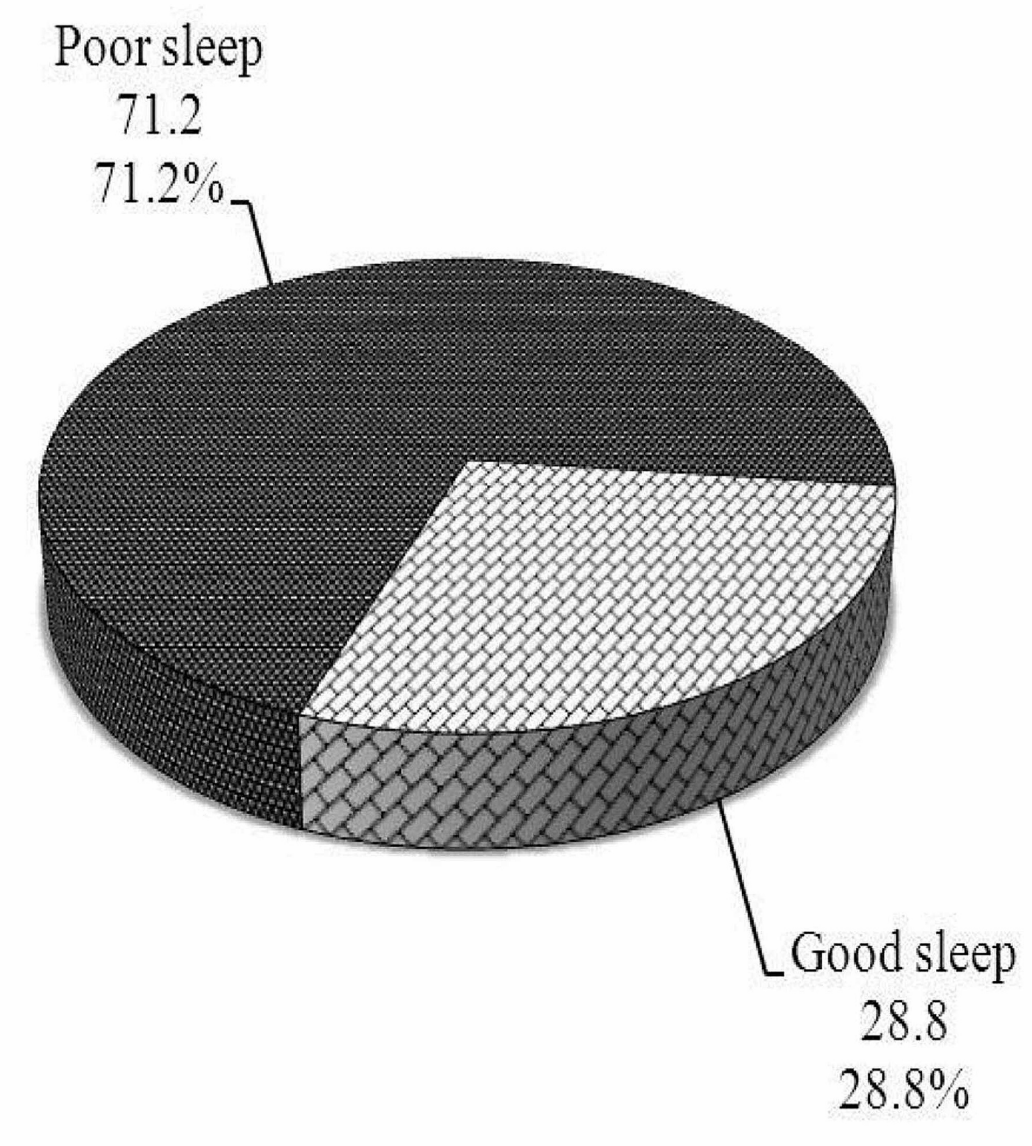
Distribution of sleep quality among the participant physicians using Pittsburg Sleep Quality Index (PSQI) (n = 344)

Moreover, sleep quality was assessed among participant physicians in our study using PSQI and revealed that poor sleep quality was present in 245 (71.2%), while good sleep quality was present in 99 (28.8%), as shown in Table 1 and Figure 3.

Our study showed significant correlations between poor sleep quality and the following parameters in the univariate logistic regression analysis: anxiety features (P value <0.001*), depressive features (P value <0.001*), and past history of COVID-19 (P value 0.003*) as shown in Table 2. However, multivariate logistic regression analysis was done among those parameters which showed significant correlations with poor sleep quality to verify which of them could be used as significant independent predictors of poor sleep quality and showed that only the presence of anxiety features (P value <0.001*) and depressive features (P value <0.001*) could be used as significant independent predictor of poor sleep quality among physicians during COVID-19 pandemic as shown in Table 2.

**Table 2:** Univariate and multivariate logistic regression analysis between the study variables and poor sleep quality among the participant physicians (n = 245/344) HADS: Hospital Anxiety Depression Scale

	Univariate		^#^Multivariate	
	p	OR (95%C.I)	p	OR (95%C.I)
Age (years)	0.411	0.986 (0.953 – 1.020)		
Gender (Female)	0.461	1.211 (0.728 – 2.015)		
Marital Status (Single)	0.930	1.025 (0.590 – 1.779)		
Weekly work hours (36 hours or more)	0.198	0.725 (0.444 – 1.183)		
Anxiety features using HADS	<0.001^*^	7.615 (4.398 – 13.183)	<0.001^*^	7.806 (3.994 – 15.254)
Depressive features using HADS	<0.001^*^	11.949 (6.909 – 20.665)	<0.001^*^	12.908 (6.915 – 24.093)
COVID 19 History	0.003^*^	3.293 (1.506 – 7.202)	0.061	2.413 (0.961 – 6.055)

## Discussion

Sleep quality is a key indicator of health as good sleep quality helps clinical staff to work better [13], is essential to boost the immunity helping to fight against the viruses and diseases [14], and to improve doctor-patient relationship [15]. Our study showed poor sleep quality among a majority of participating physicians, in agreement with a study from Iraq that revealed that working with COVID-19 patients has a negative effect on sleep among 68.3% of participating physicians [10]. Also, our results are in agreement with another study that was conducted in Bahrain, which revealed poor sleep quality among both frontline health care workers and non-frontline health care workers in 75% and 76%, respectively [5]. However, this high prevalence of poor sleep quality among our participant physicians was inconsistent with the results of Zhou et al. in 2020, which was a multicenter, cross-sectional survey conducted in Liaoning province, China that recruited 1,931 frontline health professionals. The results showed that the prevalence of poor sleep quality was relatively low at 18.4% among frontline health professionals during the COVID-19 epidemic [16]. The discrepancy in the prevalence of sleep quality in health professionals across several studies could be partly explained by different population characteristics and the assessment tools used.

Our study showed presence of anxiety and or depressive symptoms in the majority of participant physicians, in agreement with a study conducted by Lai et al. in 2020, which revealed that the psychological stress on frontline workers was incredible, compromising their sleep quality and mental health; more than 70% of health care workers in China have reported psychological distress including insomnia, anxiety, and depression [17]. Also, another study conducted by Amin et al. in 2020 showed a 43% prevalence of anxiety/depression among frontline physicians in Pakistan and alerted the need to address mental health of doctors caring for patients during this pandemic; control modifiable factors associated with it and explore the effectiveness of interventions to promote psychological well-being of physicians [18]. It was hypothesized in previous studies that several factors are associated with increased psychological distress among healthcare workers. These included fear of infecting family members and friends, uncertainty about the health consequences of the disease [19], living with children [20], being placed in quarantine [21], stigmatization, and social isolation [22].

Our study showed significant correlation between presence of COVID-19 infection history among the participant physicians and poor sleep quality and it was hypothesized in a previous study that doctors are at risk of getting infected and are often under great stress thinking of the possible transmission of the infection to their family members, colleagues, and other patients [23].

Our study also showed significant correlation between presence of anxiety and or depressive symptoms among the participant physicians and poor sleep quality, in agreement with the systemic review on the relationships among anxiety, depression, and sleep disturbance conducted by Cox et al. in 2016 that showed that insomnia and sleep quality features have “bidirectional” relationships with anxiety and depression, respectively [24]. Also, that correlation was shown in other studies which revealed that 90% of patients with depression complain of sleep disturbance [25]. Similarly, sleep problems are much more common among individuals with anxiety disorders [26]. Moreover, regarding treatment, cognitive behavioral therapy that focuses on attenuating anxiety and depression reduces insomnia severity and the symptoms of the two psychiatric conditions [27].

Limitations of the study

This was a self-administered survey that may be liable to response, recall, interviewer and social acceptability bias. Also, personality traits of the participant physicians, which might be potential predictors of mental health problems during pandemic and consequently affect sleep quality, were not included. More research and screening programs for different psychiatric features in the general population and different jobs are highly recommended.

## Conclusions

Physicians are at increased risk of exposure during pandemic infections, and therefore, they are more liable than others to mental health problems and poor sleep quality, which is relatively high. Presence of anxiety features and/or depressive features among physicians are the most significant independent predictors of poor sleep quality. Based on these findings, we recommend more interventional measures to improve sleep quality among physicians, like psychological awareness and social support during their career journey, especially during critical times like pandemics. Also, caring about sleep hours regimens quality and quantity in physician shift tables to decrease depression, anxiety, and cognitive bias during work resulting from poor sleep quality.
